# SITHON: A Wireless Network of *in Situ* Optical Cameras Applied to the Early Detection-Notification-Monitoring of Forest Fires

**DOI:** 10.3390/s90604465

**Published:** 2009-06-08

**Authors:** Georgios Tsiourlis, Stamatis Andreadakis, Pavlos Konstantinidis

**Affiliations:** 1 Forest Research Institute, National Agricultural Research Foundation, 57006 Vassilika, Thessaloniki, Greece; E-Mail: pavkon@fri.gr; 2 Telenet Wireless Communication Systems Ltd., Filellinon 25, 65302 Kavala, Greece; E-Mail: stand@telenetltd.gr

**Keywords:** forest fires, wireless network, optical camera, fire detection-notification-monitoring

## Abstract

The SITHON system, a fully wireless optical imaging system, integrating a network of *in-situ* optical cameras linking to a multi-layer GIS database operated by Control Operating Centres, has been developed in response to the need for early detection, notification and monitoring of forest fires. This article presents in detail the architecture and the components of SITHON, and demonstrates the first encouraging results of an experimental test with small controlled fires over Sithonia Peninsula in Northern Greece. The system has already been scheduled to be installed in some fire prone areas of Greece.

## 1. Introduction

Prevention, fighting and management of forest fires is perhaps the most important issue in contemporary forestry. The problem of forest fires has become of particular importance for Greece over the last decades, as increased fuel loads as a result of urbanisation and forest abandonment as well as an increased number of forest visitors have led to a rising number of forest fires and incidence in terms of fire intensity and burned area. Fire detection in Greece is based on traditional methods including permanent fire watch-points as well as patrolling fire brigade vehicles. The efficiency of those methods however, is questionable since, among other factors, they are dependent on the personal capabilities, experience and training of the personnel, which by no means they can be ensured to a high level under any circumstances. These traditional methods present difficulties to supervise large areas, to detect in a timely manner and to report the exact location of fire incidents. Despite the recent technological advances and the increased allocation of resources on fire suppression strategies, Greece still has a disappointingly high ratio of burned area per fire incident [1]. This is primarily the result of a delayed detection and subsequently of response to fire incidents. In fact, the time between the start of a fire incident and the first attack is the most crucial factor on fire suppression, thus significant efforts are required towards reducing this interval [2].

The development of automatic systems for detecting and monitoring forest fires is one of the most innovative activities in the management of forest fires [3,4]. Over the last years several systems for forest fire surveillance and detection from ground or satellites have been developed. Satellite systems, such as the Fire M3 system (Fire Monitoring, Mapping, and Modelling) [5], appear to be more efficient in monitoring large forest areas like those found in Canada. Such systems are currently under development for Europe as well, including FUEGO [6] for detecting forest fires and RISK-EOS [7] for mapping burned areas. At the same time, various types of detectors and cameras have been investigated for their practicability in the development of ground fire detection systems, like CCD (Charge Coupled Device) sensor data [8], infrared detectors (IR) [9], detectors like LIDAR (LIght Detection And Ranging) [10], and RADAR [11]. The RADAR can be used to detect turbulences created in the column of air above the fire, while the LIDAR-type detectors emit laser beams to detect the constituents of smoke. Despite the effectiveness of these techniques, the high costs of further research and for maintenance make them difficult to operate on a large scale [12, 13]. Recently, the application of a radio-acoustic sounding system (RASS) to create thermal maps of forests areas for detection of potential fires was proposed in Turkey [14]. Infrared sensors are used detect hot spots (temperature changes) and are therefore suitable for fire detection systems. Several commercial systems, such as BOSQUE (IZAR-FABA) and BSDS (Teletron) [15, 16] are based on detection in the thermal infrared (TIR) part of the electromagnetic (E/M) spectrum. They are easy to use and the cost, while not prohibitive, is not cheap either (25,000 Euros). However, the majority of fires start in areas that are hidden by trees and other objects. Consequently, the infrared detectors on terrestrial fixed platforms detect fires only when they reach the top of trees or surpass obstacles. Optical cameras using CCD sensors (chips) operating in the visible range, on the other hand, are very cheap (2,500 Euros). They can be used to visually scan large areas and they operate under all weather conditions. Their disadvantage is that the detection is only visual and is based on to the existence of a smoke column. The sharpness of the image decreases substantially with the distance of observation and for this reason the installation of a network of detectors is usually required. Nowadays, in several countries, different terrestrial surveillance and automatic forest fire detection systems, based on CCD video cameras sensitive in visible and near IR spectra are operating and certain of them provided commercially. Examples are FireWatch (Germany) [17], FireHawk (South Africa) [18], ForestWatch (Canada) [19], UraFire (France) [20], IPNAS (Croatia) [21], etc. All these systems have in common that the fire detection is based on smoke recognition during the day and flame recognition during the night.

In the frame of the Greek project SITHON, two methods for the detection of forest fires, one in the visible spectrum and one in infrared have been tested and evaluated regarding their capabilities for early detection and monitoring. A detailed description of the airborne thermal imaging components of the SITHON system is already available in a previous issue of the journal [22]. In the present paper, the architecture and the components of the wireless network of *in situ* optical cameras are described in detail, followed by a brief description of the results of field tests and conclusions.

## 2. The SITHON Forest Fire Detection System

### 2.1. Overview

The main objectives of the SITHON project were to use both terrestrial and airborne technologies to achieve an effective detection, monitoring and management of forest fires. The effective detection goal included a geo-referenced automatic high accuracy location of the fire event and a considerably reduction of event notification at the level of several minutes, in order to provide a substantial improvement in comparison with traditional methods. The second specific goal was the ability to efficiently monitor and survey large forested areas, day and night. Finally, to provide continuous information in order to facilitate the management of forest fires and improve the coordination of fire-fighting forces and all services involved.

Another important goal was to provide an autonomous low cost system fully adapted to the particular mountainous morphology of Greece, where forested areas usually consist of a mosaic of many natural ecosystems at different structural levels. The SITHON system is the product of a research project, which started five years ago, when market systems based on optical CCD video cameras were still under development in countries with totally different environmental conditions (mainly non- mountainous relief and uniform vegetation). Moreover, these systems were and still are quite expensive if they have to be adapted to the Mediterranean environment. Further, they are still based on simple topographical information layers usually already available.

**Figure 1.**
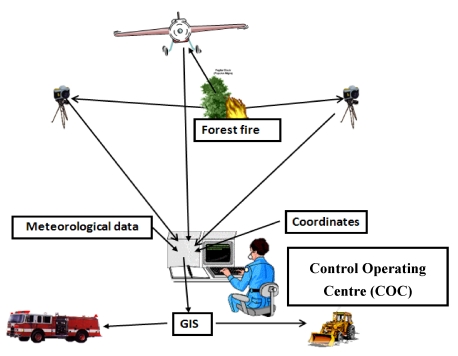
Description of the SITHON system.

The SITHON system consists of a wirele**s**s network of *in situ* optical cameras, and an airborne fire detection system based on a fully digital thermal imaging sensor (Figure 1). The network of optical sensors consists of monitoring towers, transmitters and wireless transmission units, linked to an integrated GIS environment in order to facilitate the fire fighting management and support the decision making process during forest fires. The GIS database incorporates qualitative and quantitative information layers necessary for the estimation of fire risk. This includes information about the vegetation types, fuel load quantities, the road network for accessing active fires, the area's morphology, high risk locations (settlements, camps, folds, archaeological sites, etc.), sensitive infrastructures (fuel stations, flammable materials, industrial areas, etc.), availability of natural or artificial water reservoirs and more. The SITHON platform includes a Control and Monitoring Centre or Control Operating Centre (COC), which receives information in the form of optical and thermal images from the wireless sensor detection systems. The optical images are displayed on wide screen monitors and analyzed to derive the dynamic picture of fire evolution.

Networks of cameras operating in the visible spectrum may be used to monitor large areas and they can be operated by a single user creating suitable conditions for a reliable monitoring. In the chosen experimental area of the Sithonia Peninsula, the appropriate locations for the installation of the optical cameras were selected taking into account technical needs and the geomorphology of the terrain. Further aspects were the fire hazards of the particular vegetation types, the temperatures prevailing during the summer months and the intensity and direction of local prevailing winds. The locations chosen provided the best possible supervision of the area and they had direct visual contact to at least one neighboring camera station to set up the network structure. The whole system was designed to communicate through the wireless network with the COC, which was established at the “Agricultural Bee Keeping Cooperative of Nikiti–Chalkidiki”.

The devices and the fully digital integrated sensors used had as main characteristics a wireless data transfer (between the remote cameras and the COC and *vice versa*), an interoperability and interlink with mobile devices and the internet, a real time and live data retrieval, and a save and secure data transfer. In the following sections a detailed description of the wireless network of *in situ* optical cameras part of the SITHON system is given.

### 2.2. Wireless network architecture

The wireless technologies are implemented with “common access way”, which means that all terminals compete at all times for access to common means of data transmission. There are two basic topologies that can be applied: the “point-to-multipoint (PtM)” and the “point-to-point (PtP)”. In the PtM, one or a few central spots (PoP) control all the other terminals. This topology requires only a small amount of active network equipment and therefore the network is easier to construct and manage. Conversely, the total bandwidth in each sector is split between the terminals and the total amount of transferred data is reduced. In the case of PtP topology, the interface of each site is point-to-point linked with the central point or through an intermediary. Consequently, the available bandwidth is the entire range that can provide a wireless connection. It requires a larger number of networked wireless devices and the network is complex to manage. However, the network presents better reliability and system stability (no general breakdown if one central point breaks).

**Figure 2.**
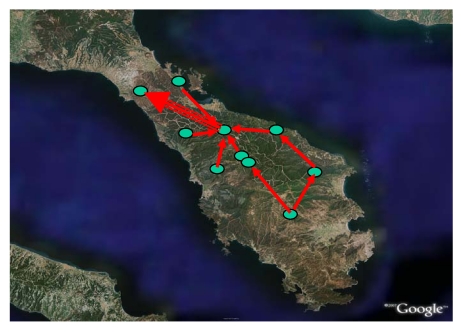
Location and links of terminals – optical cameras (Sithonia, Greece).

For the telematic network of Sithonia (Figure 2), the Point-to-point topology was selected as the best solution given the mountainous nature of the terrain and the amount of data to transfer. The network consisted of one main terminal (also equipped with a camera), where the COC was installed, ten terminals (with cameras) and four transmitters. The wireless connections were implemented using only protocol Wi-Fi 802.11a in the frequency range 5.1-5.8 GHz. The network of *in situ* optical cameras covers a total mountainous area of 45,000 ha, which consists of Alleppo and Black pine forests (40% and 10% respectively) in the north-central part of the Peninsula, Mediterranean type shrublands (20%) in the East, phrygana (Greek low dwarf-shrub vegetation, 20%) in the South, and cultivations (olive groves, vineyards, etc.) and urbanized areas along coasts.

**Photo 1.**
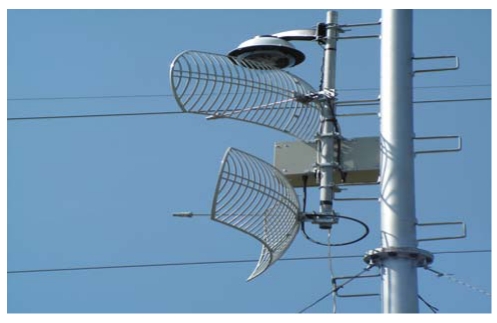
Optical camera with protection cover, antennas and box with the point to point backbone bridge.

The specifications of the antennas were determined based on secure communication between links depending on the least possible charge on pillars due to variable tension forces exerted by the air on their surface. Their gain was also counted in the low final value of EIRP (effective radiated power) of the data transmission system. The wireless network consists of the distribution segment, which links the ends of the backbone network with the cameras. For the connection of the access points to the network backbone, parabolic “mirrors” (Photo 1) were used for the communication links “point to point” both for the backbone network and the distribution network. With the use of the specific antennas, the maximum directionality of the microwave transmission was attained, saving energy, while surrounding areas were not charged with unnecessary radiation.

**Figure 3.**
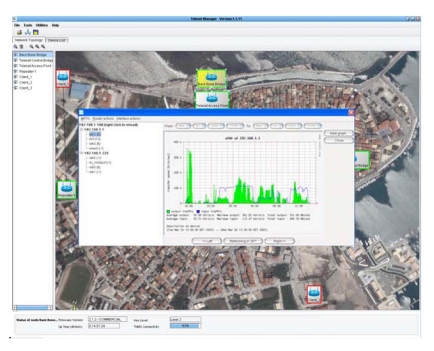
Multi-router traffic monitor of the network data on each link separately.

Within the frame of the project, a powerful tool (Telenet Network Management System, “TNMS”) has been developed for the management of integrated wireless networks and their devices. The TNMS allows the remote network control, data management, and system control. The topology diagram, live video images from all the *in situ* cameras, as well as statistics of the whole network are displayed in real-time. The control of the network is achieved by remotely adapting the settings of the devices, sending automatically malfunction messages and resetting them if necessary. The system has the ability to monitor the network data on each link separately (traffic monitor) as it is illustrated in Figure 3 and to adjust the volume data.

The system is controlled by displaying signal and noise levels, and the quality of each link, and operating the frequency and the emission power of each device and antenna. The TNMS contributes to a secure function of the network by allowing the change of the encryption way and the codes of mobile data and providing alerts in case of a closure device or an attempt of violation of encryption codes. The advanced technologies applied, allows the creation of reliable and low-cost networks that operate at frequencies that do not require special permits to install and frequency use. At the same time, the low energy consumption and radiation microwave devices, contribute to the implementation of an autonomous model of communication, ensuring extremely low function cost.

### 2.3. Self-supporting monitoring (camera) tower

**Figure 4.**
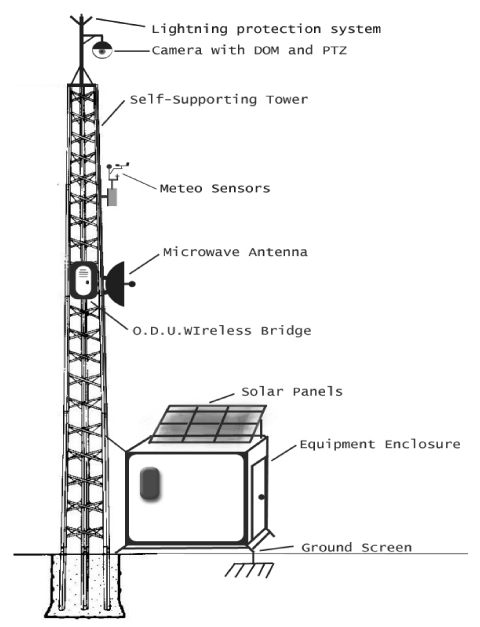
Self-supporting tower subsystem and monitoring sensors.

The subsystem consists of monitoring, transmitting and energy supporting devices fixed on a pillar plus enclosure equipment (Figure 4, Photo 1). The main characteristics of the devices are described in the followings paragraphs and in Table 1.

The monitoring devices comprise an optical camera that captures high resolution images with a powerful zoom in real-time. The camera is digitally controlled by a pan tilt step motor unit that allows horizontal and vertical rotation. The pan tilt step motor has the ability to memorize pre-located points and also provides automatic (repeated cycle movement at preset points) or manual scan of the camera. A waterproof protective cover (Dome) protects the camera. The system allows the installation of collection units of meteorological data with the device transmitting data.

**Figure 5.**
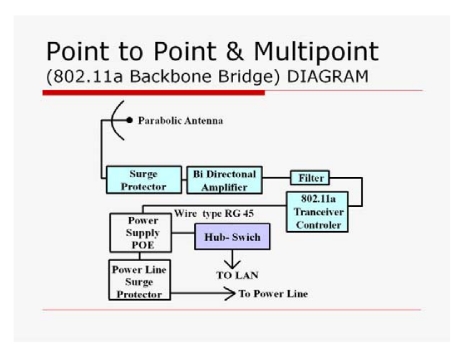
Point to point Backbone Bridge diagram.

The transmitting system (Figure 5) includes the active and passive telecommunications equipment, which provides the ability of creating a wireless network for broadband data transfer. It comprises microwave passive parabolic antennas, bi-directional amplifier, hub-switch and a secure supply system. A camera server converts in real-time the analog signal of the camera do a digital video stream.

The geographical characteristics of the area required the use of four pillar transmitters at points where there was no visual contact in the network between pillars or the Centre. The subsystem consisted of a self-supporting tower, a switch – hub, a solar energy system, an equipment enclosure, a lightning protection system and the necessary subsystem software.

The pillar is a self-supporting tower, which has a set of standards that, first, ensure maximum static efficiency of the devices with minimal vibration and the resistance to extreme weather conditions. The final height was determined based on sight ability and the minimum environmental disturbance of the site. The selected locations are frequently affected by extreme weather events. This imposes the need for installation of lightning protection systems. Solar panels provided power to the monitoring towers without possibility of connection to the electricity network.

### 2.4. Control operating centre

The Control Operating Centre is the reception room where the final analysis of the information from the *in-situ* cameras is done. It has been designed to function at prefectural Fire Brigade Departments, which, in Greece, are responsible for the surveillance and protection of forests from fires. The COC controlling a system of the describe size (test) is organized in a way to be handled by two or three operators, scientific staff from the Fire Brigade. The operators have to be trained to the techniques used, especially those involving handling of the GIS data base.

**Photo 2.**
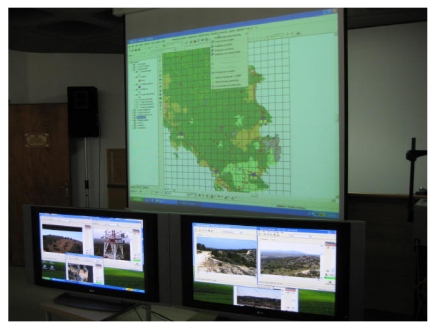
Crisis Operating Centre with large plasma and soft screens, where the live video-streams from the *in situ* cameras and the GIS data base were respectively displayed.

The Centre was equipped with two large plasma screens (42″) that allow the observation of the area and display information about the network status to one or two of the operators. The operator(s) can manually control the cameras and zoom in-out in order to get information about a fire. The second or third operator elaborates the GIS data base and provides continuous information (available also in projection) needed to facilitate the fire fighting and support the decisions of the Officer or Coordinator in charge (Photo 2). The communication with the terrain/executive forces is made by VHF transmission, as it is the practice of the Fire Brigade. However, in the future, according the acquisition of new equipment by the Fire Brigades, it could be also done using the wireless network (e.g. fleet control, VOIP, direct surveillance on lap-tops in the terrain).

The Centre was equipped with a suitable computer system consisting of a data base server and data server, web server, GIS and application server. The software included monitoring software for the wireless network status and the flow rates of the data link network; monitoring and management software of the devices for the automatic control of autonomous power units at the remote parts of pillars. Moreover, several components were included to ensure security of the network and the database by potential intruders (hackers): a firewall and a proxy server assumed the role of shielding the internal network. The safety of data transferring was provided using a digital protocol SSL 128 bit encryption. The safety of archive was provided by a backup unit, which could save the contents of the database at regular intervals. The maintenance of the whole system has to be achieved by specialists in wireless telecommunications and GIS.

## 3. Automated Detection of Fire

Software, still in development, allows the automatic detection of smoke or fires based on the analysis of identical images, which the system takes at pre-located points, memorized by the tilt step motors, when the cameras automatically turn with a full horizontal rotation in steps of 10° or 5°. The comparison with stored images reveals differences in pixels and also matches with the predefined criteria of the properties of smoke and fire. The system automatically detects smoke or fire and provides a sound alarm, the time record, the camera and the angle (azimuth), where the incident occurred. Immediately, the operator focuses on the event, with live video, assesses if it is a false alarm (such as controlled fire from farmers, dust from cars) and processes the data in the control database. The image processing consists of comparing the reference image to the current image, using normalization, filtering, matching, and cluster search algorithm and probability assessment. Non-linear filtering of the reference image suppresses any movement. Thresholds are formed proportional to normalized standard deviations. The images are compared to produce a binary difference image using the thresholds. A cluster search algorithm is used to locate related areas. The image processing computer uses complex algorithms to identify smoke in real time.

Once the identification is given by a second camera, the coordinates are automatically determined in the database based on trigonometric calculation. In any case, until the fire is detected from the second camera, the operator can determine the coordinates manually, using fixed ground control points such as the shoreline, roads, peaks, etc.

## 4. Public Acceptance and Awareness

One of the main concerns prior to the installation of the network was to avoid alienating the local community due to the intrusive nature of the project (supervision of areas with cameras). Thus, an extensive campaign was undertaken before the start of the project in order to inform both local authorities and the public on the potential benefits of the project. As a result there was no systematic opposition, and in fact, both authorities and the main users of the forest (apiculturists) showed a great interest and contributed in practice to the implementation of the network in the following issues:

– Ensuring the permits for the installation of pillars in forest areas.
– Providing electricity where it was possible.
– Provide space to install the COC.
– Ensuring the permission for the experimental fires.
– Ensuring adequate quantity of combustible material in selected places.

Another concern was the emitted electromagnetic fields. The international protocol Wi-Fi 802.11a was chosen as the method for transferring data mainly because of its free access and its low-power communication, necessary to overcome concerns of local inhabitants related to the emitted radiation. The signals are very low power in both the computer and the router (access point) and the results so far show that exposures are well within internationally accepted ICNIRP guidelines [23] and in accordance with the applicable laws [Law 3431, 13/A/03-02-2006 GG]. It is worth mentioning that in the type of antennas used the radiation beam is super-directional and the maximum radiation direction is extremely unlikely to meet points accessible to the public, given a possible interference of natural or artificial barriers making the connection problematic.

At the end of experiment, there were two days of exhibition of the SITHON system: one for the Media and another for the Regional Services involved in the fire prevention and fighting, such as the Fire Brigade, Forest Services and Municipalities.

## 5. Experimental Setup of the Field Tests

**Photo 3.**
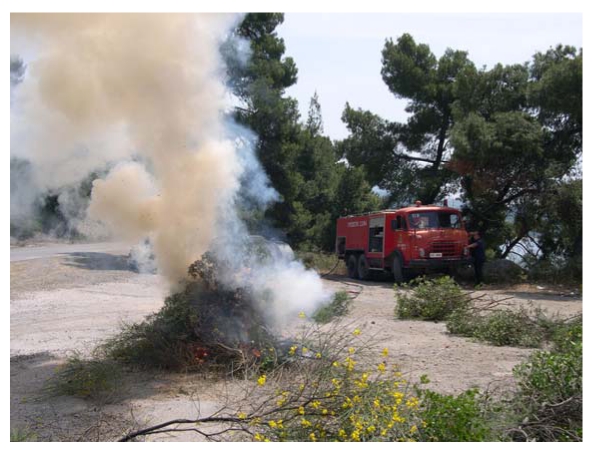
Experimental fire.

In order to assess the potential and the operational ability of the system an experiment took place between 2 May 2005 and 15 June 2005. For calibrating the system and checking its reliability, suitable areas were chosen and prepared in order to create low intensity controlled fires (±3 m in diameter, Photo 3). These sites covered the entire peninsula, with emphasis of the more forested areas, and they were located either on firebreaks and road sides, or in forest gaps. At each site fuel material collected from branches of pine trees and local shrubs was accumulated. Special attention was paid to ensuring accessibility by fire brigade vehicles on each site in order to avoid potential spread of the experimental fire on the nearby forested areas. The local fire Brigade and the Municipality provided a sufficient number of fire brigade vehicles and the appropriate staff for the entire duration of the experiment.

The period of calibration took a week. During this period the scientists responsible for the program and all other technicians evaluated any problems encountered. A debriefing was organized at the end of each day to assess the level of preparedness and to discuss problems and future tasks.

**Figure 6.**
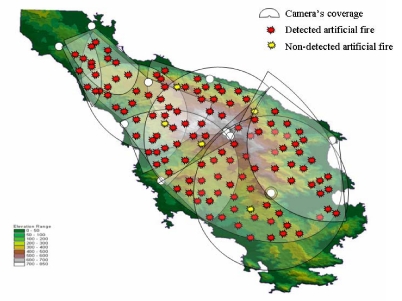
Cameras' coverage and locations of artificial fires.

The design allowed for causing 4-5 fires every day at different locations or times (between 9 AM and 3 PM). Following a strict protocol (data of artificial fire, weather conditions, tracking timing, *etc.*) the allowable levels at which fires could be occurred were fixed. Thus, for safety reasons, days with winds exceeding 5 Beaufort had been excluded. The Associate Scientific Responsible of the program took over the responsibility for the safe execution of the experiment outdoors and the Scientific Responsible was in charge in the Control Operating Centre. We mention that, according the strict scientific protocol, both were the only ones who knew these locations and the time of ignitions. Finally, it was possible to make 95 repetitions (Figure 6).

It is worth mention that the difficulty to get permission to perform tests at this scale brings a special value to the whole experiment.

## 6. Results and Discussion

During the period of field tests, the ten cameras were moving automatically horizontally. The operators were monitoring the real-time video live-streams on two large plasma screens (Photo 2). Despite the full rotation ability of cameras, they were programmed to monitor only sectors of the terrain, in a way at least two cameras are monitoring each point of the area (Figure 6). This fact allowed the camera to carry out their movements more quickly and therefore to perform earlier detection. However, the south part of the peninsula, where the low sparse phrygana vegetation dominated, was monitored by only one camera in order to reduce the total cost. The revolution range was between 90 to 210 degrees (except the southwest camera that had a full rotation) and the depth of surveillance 5 to 15 km. It appears the optimum coverage range detection reached 10 km, value that is sufficient in the local morphological conditions, which are comparable with the majority of the mountains of Greece. It is a comparable value regarding the market available FireWatch [24].

Once smoke was detected, the operator manually zoomed in on the event in order to determine more precisely the nature of the incident. In a specific protocol several data were stored including: the location (coordinates, Grid, place-name) of ignition, the position of the nearest pillar; the time of ignition, the time of detection by the cameras; the time of detection from a 2^nd^ camera and its distance from the ignition point; the weather conditions (wind intensity and direction), air temperature, relative humidity, visibility); fuel type; and the time of processing. From the 95 repetitions, 72 (75.8%) were made under perfect visibility, 12 (12.6%) under moderate (8-12 km perfect visibility) and 11 (11.6%) under low visibility conditions (perfect visibility <8 km). Although the main transmitters based their power supply on solar energy and were several cloudy days, the network worked perfectly during the experiment.

Table 2 shows the response of the cameras network to detect the fire event per class time. Two thirds of the repetitions (62/95) were detected from at least one camera in less than two minutes from the ignition, while the 90 fires in total were detected within ten minutes of ignition. Detection from a second camera within less than two minutes occurred in 43% (41/95) of the cases. This performance could be considered as very good in absolute value of time detection. Moreover, despite the fact these are results of an experiment, which has size restrictions in the fire tests, they are comparable to FireWatch. Its official website [24] announced a time alert of 4 or 2 minutes in the case of a single or multi tower system respectively.

The four fires which were not detected at all by a second camera, all occurred on the east coast, where morphology made difficult the installation of the cameras in the more appropriate locations, but in these cases, the coastal line helped to identify manually the fire coordinates. It was observed that the great majority of cases were detected in less than five minutes by either one camera (85%, 81/95) or two cameras (80%, 76/95). It is noteworthy that 38% (36/95) of the ignition point coordinates were calculated automatically within the first two minutes and 78% (74/95) within the first five minutes. The system had detection accuracy during the experiment, independently of the visibility, of 95% of fire events of 3 × 3 m in 10 km. The FireWatch system has an automatic detection accuracy of 100% of smoke clouds of 10 × 10 m in 10 km [24].

It was estimated that the detection time was proportional to the altitudinal difference between the ignition point and the ridge. We estimated empirically that for every 50 m altitudinal difference, the delay was about 30 seconds. However, five of the fires were not detected at all (Figure 6). These fires were situated in deep gorges and the wind speed was about 4-5 Beaufort. For that reason the smoke diffused before reaching the field of view of cameras. This failure is most likely due to the bad conditions for the observations of such small fires (according to the protocol, for safety reasons) and therefore only little smoke instead a real fire incident. At the same time, there were several false alarms due to cases not related to the experiment. Three cases involved burning olive groves branches and two cases of dust from cars moving at high speed on forest roads.

The wireless network functioned without any problems. The remote control of the whole system was effective, the automatic movements of the cameras reliable and the manual handling of the cameras relatively easy and efficient. The quality of the video live-streams was excellent, except under rainy conditions and low clouds. We have to mention that two more cameras would improve the detection ability in the Southwest and South parts of the peninsula, but as presented above, the lower risk of real fire event in these areas and economical reasons limited the size of the network. It appears the system presents a relative sensitivity to very strong winds (≥ 7 Beaufort). Therefore, it is a need to reinforce the metallic structure of the towers. A third large plasma screen would be useful in order to monitor a maximum of four cameras in each monitor. Moreover, despite theoretically one observer could handle the system, it is better to have two observers sharing the monitoring. One of the advantages of the system proposed is that only one computer system is controlling the whole network of ten cameras. It is located at the COC avoiding potential vandalisms or destruction by natural hazards. In the contrary usually market systems have computers that centralize data from 4-5 detection-surveillance sites and data processing and analysis equipment is based at the vision-detection towers [17, 24].

The following year, a restricted application of a first version of specific software for automatic smoke or fire detection (see Section 3) was locally tested in the area near the COC with encouraging efficiency, improving the usability of the system SITHON. However, it displayed several problems under strong wind conditions. The software, which is still under development, will be tested more intensely in the future, once an extensive experiment is allowed.

## 7. Conclusions

The SITHON system has used broadband technology to create a wireless network of *in situ* optical cameras that provides early detection and notification of forest fires and continuous live monitoring of large fire-prone regions. The SITHON system introduces in Greece new innovative technologies for fire detection, allowing the continuous monitoring of the incident aiming to improve the efficiency of fire fighting. The results of the current experiment were more than encouraging in comparison with the fixed goals and moreover.

A significant reduction of fire detection time was achieved, reaching less than 2 minutes for the majority of cases, as well as an automatic calculation of the fire ignition point coordinates within less than 5 minutes in most cases. It is reasonable to assume that under real conditions the system will be even more efficient, since the smoke in these cases is much more abundant and therefore easier to identify.

The system is an independent digital surveillance (automatic scanning) system that is able to observe large areas and analyze, associate and store the collected data (images, meteorological data). In case of a fire event, an operator or the system automatically sends an alarm and the system provides the coordinates of the incident. Meanwhile, the network of sensors is linked to a GIS data base that provides relevant information for an efficient estimation of the fire risk, fire fighting and incident management, such as the vegetation types and quantity of fuel, the road network for accessing the active fires, the area's morphology, the sensitive and endangered locations, etc. An automated subroutine data-processing data modeling provides immediately the fastest track to access the location of fire. The live stream capabilities of the system enable the Control Operating Centre to have always the real “live” picture of the incident and aiding significantly the decision making process. Alongside the broadband network can provide great services to the wireless communication between the agencies forces involved in fire fighting. It appears the usability of the system will be significantly improved with the incorporation of the automated smoke and fire detection software. However, this has to be combined with reinforcement the monitoring towers in order to increase the stability necessary when faced with strong wind conditions.

Despite the official permissions received to install the network and to conduct such a specific experimental field test, the team took into account very seriously the concerns regarding public acceptance and awareness. The Wi-Fi 802.11a protocol was chosen because the extremely low electromagnetic fields emitted and the super-directional type of antennas required. Moreover, local authorities and the important community of apiculturists, as the main users of the forest, contributed to the implementation of the network. The system was presented to the Fire and Forest Services of the region, who assessed both its efficiency and usability. In a first phase, the GIS data base has been installed at the two Services. There was also a very good acceptance from local and national media and as a result, the system became widely known throughout the country. There was considerable interest in many regions for the system. The low cost of the proposed system (at the third to half of the price of comparable commercially available systems) in combination with its efficiency and its general acceptance convinced the National Authorities of its great potential for the protection of fire prone forests of Greece. The system has already been scheduled to be installed in some other fire prone areas of Greece.

Moreover, the SITHON system contributes to the overall protection and enhancement of environment. In combination with existing fire suppression strategies, the installed cameras scan the most ecologically sensitive areas of the monitored zone, and areas of possible infringement. Also, using the capacity of the wireless network, images from additional cameras could be available via the Internet around the world, highlighting the ecological values of the region.

With the full operating system SITHON the following objectives were achieved:

– A continuous effective monitoring of large forested areas.
– A prompt detection of a fire event followed by an immediate notification to the Fire Brigade.
– An accurate location of the fire event, facilitating the coordination of fire-fighting forces for more efficient and fast transfer to the event.
– Continuous provision with information for optimal coordination of fire fighting.
– A reduction of the number of personnel employed to supervise the area.
– An effective psychological effect on both potential arsonists and users of the forest in general.
– A direct and precise identification of potential infringement (illegal grazing, illegal logging, etc.).
– Recording of ecological disturbances and possible changes in the natural environment.
– Transmission of important information to all interested Services in real time.

## Figures and Tables

**Table 1.** Monitoring tower's devices technical characteristics.

**Equipment**	**Technical characteristics**
Optical camera	High resolution real-time up 25 images per second. Mechanical zoom 22 X. Pal video signal; 470.000 pixels; S / N ratio: 48 db; Focal 1,4 to 2,8; Auto or Manual Focus; Auto iris servo system aperture; Pan angle range: ± 170°; Tilt angle range: + 10° −90°; Pan / Tilt rotation speed: Pan = 1 to 90 deg / sec Tilt = 1 to 70 deg / sec; RS232C: 2 min-Din 8 pin; Power supply: 220 - 240 V AC; Power consumption: 12W. Voltage: 13 V DC.
Pan Tilt step motors unit	Rotation: 350 degrees horizontally and 100 degrees on the vertical axis. Position accuracy: 5 / 100 degree. Memorisation accuracy: 1/1,000 degree. Movement: automatic and manual.
Camera Dome	Watertightness «IP65» standardization.
Collection units of meteorological data	Air temperature, wind speed, wind direction, relative humidity and barometric pressure. TCP/IP data transfer protocol. RJ45 port.
Camera Server	Composite video to video stream real-time conversion (min. 25 frame/sec) following TCP/IP protocol.
Point to Point Backbone Bridge	Specific microwave passive parabolic antennas with high gain directional emission. External O.D.U. Wireless Bridge: Bi-directional amplifier transceivers (both transmission and reception) with low power emission (limit of 100 miliwatt). Lightning components (Gas Tubes) and transit zone filters in order to avoid any kind of interference. Supply system through Ethernet port (P.O.E.) used to prevent high voltage transmission in the pillar.
Hub-switch	Standard transit data, 19 in. rack mount, 10-100 Mbps, min 8 ports RJ45.
Pillar (Self-Supporting Tower)	Latticed and self-grounded heavy type. 6–12 m height.
Wall Mount Cabinet	Metal construction, standard size 19 in. in size at least 16 U, with a glass door, thermostat and fans.
Equipment enclosure	High standard metal construction framed with steel polyurethane panels electro-statically painted, complete electrical installation, and ventilation with air filters and fan.
Solar Energy System	One-crystal silicon solar panels, dry type batteries, automation load control and energy efficiency with remote management, converter / inverter DC 24 V - AC 220 V. Autonomy of 72 h.
UPS (Uninterruptible power systems)	Maintenance capacity of the system for up to 8 h.
Lightning Protection System	Type Franklin, with a full equipment load transmission lightning and earthing grid.

**Table 2.** Detection of artificial fire events from a first and second *in situ* camera and definition of its coordinates by response time class.

**Response Time (in minutes)**	**Events detected by a 1^st^ camera**	**%**	**Events detected by a 2^nd^ camera**	**%**	**Definition of event's coordinates**	**%**
1-2′	62	65.3	41	43.2	36	37.9
2-5′	19	20.0	35	36.8	38	40.0
5-10′	9	9.5	10	10.5	12	12.6
Total identified events	90	94.7	86	90.5	86	90.5
					4*	4.2
Total non-identified events	5	5.3	9	9.5	5	5.3
Total events	95	100.0	95	100.0	95	100.0

* Manual finding
